# Diagnostic Accuracy of the Patra Index and Red Cell Distribution Width Index in Distinguishing Beta-Thalassemia Trait From Iron Deficiency Anemia: A Retrospective Comparative Study

**DOI:** 10.7759/cureus.104454

**Published:** 2026-02-28

**Authors:** Satyabrata Patra, Abhirup Shome, Camellia C Patra, Sunil Kumar Mahto

**Affiliations:** 1 Pathology, Rajendra Institute of Medical Sciences, Ranchi, IND; 2 Anatomy, Rajendra Institute of Medical Sciences, Ranchi, IND

**Keywords:** beta-thalassemia trait, iron deficiency anemia, mean corpuscular volume, patra index, red cell distribution width, red cell distributionwidth index

## Abstract

Background

Distinguishing beta-thalassemia trait (BTT) from iron deficiency anemia (IDA) remains a clinical challenge, particularly in resource-limited settings where nutritional anemia is highly prevalent. This study aimed to compare the diagnostic accuracy of the Patra index with the red cell distribution width index (RDWI) for differentiating BTT from IDA, using high-performance liquid chromatography (HPLC) and serum ferritin as reference standards.

Methods

This retrospective diagnostic accuracy study was conducted at Rajendra Institute of Medical Sciences, Ranchi, India. A total of 156 patients diagnosed with either BTT or IDA were included. BTT was defined as hemoglobin A2 (HbA2) > 3.5%, and IDA as serum ferritin < 15 ng/mL with HbA2 < 3.5%. The Patra index was calculated as mean corpuscular volume divided by red cell distribution width (MCV/RDW), with a cutoff of ≥5.3 for BTT. RDWI was calculated with a cutoff of <220 for BTT. Diagnostic performance metrics, including sensitivity, specificity, positive predictive value (PPV), negative predictive value (NPV), and 95% confidence intervals (CIs), were determined. Receiver operating characteristic (ROC) curve analysis was performed to assess overall discriminative ability.

Results

Of the 156 patients, 53 (33.97%) had BTT and 103 (66.03%) had IDA. At the predefined cutoff, the Patra index demonstrated a sensitivity of 83.02% (95% CI: 70.2%-91.9%) and a specificity of 100.0% (95% CI: 96.5%-100.0%), yielding a diagnostic accuracy of 94.23%. In comparison, RDWI showed a sensitivity of 100.0% (95% CI: 93.3%-100.0%) and a specificity of 81.55% (95% CI: 72.7%-88.5%), with a diagnostic accuracy of 87.82%. The area under the ROC curve for the Patra index was 0.989.

Conclusion

In this study population, the Patra index exhibited high specificity for differentiating biochemically confirmed BTT from IDA, although its moderate sensitivity limits its utility as a standalone screening tool. It may serve as a simple, cost-effective adjunct or triage tool in resource-limited settings, with confirmatory biochemical testing remaining essential for definitive diagnosis.

## Introduction

Differentiating beta-thalassemia trait (BTT) from iron deficiency anemia (IDA) remains a longstanding diagnostic challenge in routine clinical practice [1]. This issue is particularly pronounced in populations where both malnutrition-related IDA and BTT are prevalent, as patients often present with overlapping hematological features [2,3].

High-performance liquid chromatography (HPLC) remains the gold standard for diagnosing BTT, while serum ferritin is the reference standard for IDA [4]. However, ferritin interpretation may be confounded by inflammatory states, and both HPLC and ferritin testing are often unavailable in resource-constrained settings, including many district-level hospitals [4,5]. Consequently, clinicians in these settings frequently rely on complete blood count (CBC) parameters and derived red cell indices for preliminary evaluation [5,6].

Among these indices, the Mentzer index is the most widely used globally, followed by the red cell distribution width index (RDWI) [7,8]. Nevertheless, several studies have demonstrated that the diagnostic performance of these indices is limited in populations with a high prevalence of nutritional deficiencies, where patients may exhibit features of both BTT and IDA. This compromises their utility as standalone screening tools [5,6,9].

Given these limitations, there is a need to evaluate simpler, readily accessible indices that maintain acceptable diagnostic accuracy in resource-limited settings with overlapping BTT and IDA features. The Patra index, defined as the ratio of mean corpuscular volume (MCV) to red cell distribution width (RDW), has been proposed as a potential alternative. However, evidence regarding its performance remains limited.

The present study aims to compare the diagnostic accuracy of the Patra index with RDWI in differentiating BTT from IDA, using HPLC and serum ferritin as reference standards.

## Materials and methods

Study design

This hospital-based, retrospective diagnostic accuracy study was conducted to evaluate the performance of the Patra Index and RDWI in distinguishing between BTT and IDA. 

Study setting and duration

The study was carried out in the Department of Pathology at Rajendra Institute of Medical Sciences, Ranchi, India. Patient records were retrospectively reviewed over an 18-month period to ensure sufficient case accrual for diagnostic performance analysis and receiver operating characteristic (ROC) curve evaluation.

Study population and eligibility criteria

Patients evaluated for microcytic anemia and diagnosed with either BTT or IDA based on predefined reference standards were included. BTT was defined as hemoglobin A2 (HbA2) > 3.5% on HPLC, while IDA was defined as serum ferritin < 15 ng/mL with HbA2 < 3.5%. Cases meeting both biochemical criteria were excluded to maintain mutually exclusive diagnostic groups. Additional exclusion criteria were incomplete records, ongoing empirical iron therapy, and lack of informed consent.

Sample size

As a retrospective study, no formal a priori sample size calculation was performed. All eligible cases during the study period were included, resulting in a total of 156 patients: 53 with BTT and 103 with IDA. Detailed demographic and clinical variables, including age stratification, pregnancy status, inpatient versus outpatient classification, comorbidities, and recent transfusion history, were inconsistently available and therefore not included in the analysis.

Ethics statement

The study was approved by the Institutional Ethics Committee of Rajendra Institute of Medical Sciences, Ranchi, India (ECR/769/INST/JH/2015/RR-21; Letter No. 132, Dated: 10/04/2024). Patient confidentiality was maintained throughout the study.

Laboratory parameters and index calculations

Peripheral blood samples were collected in EDTA vials and analyzed using a Horiba Yumizen H2500 CBC analyzer (Horiba, Ltd., Kyoto, Japan). The recorded parameters included total red blood cell (RBC) count, MCV, mean corpuscular hemoglobin (MCH), hemoglobin (Hb), mean corpuscular hemoglobin concentration (MCHC), and red cell distribution width (RDW).

The Patra Index was calculated as MCV ÷ RDW. The RDWI was calculated as (MCV × RDW) ÷ RBC, with a predefined cutoff of <220 for BTT and >220 for IDA [7].

Reference standard

Index-based diagnoses were compared against the reference standards. BTT was confirmed by HPLC (HbA2 > 3.5%), and IDA was confirmed by serum ferritin < 15 ng/mL with HbA2 < 3.5%.

Statistical analysis

For each index, sensitivity, specificity, positive predictive value (PPV), negative predictive value (NPV), and corresponding 95% confidence intervals (CIs) were calculated. ROC curve analysis was performed, and the area under the curve (AUC) with 95% CI was estimated to assess discriminatory performance at the predefined cutoff values.

## Results

A total of 156 patients were included in the study. Based on HPLC and serum ferritin measurements, 53 patients (33.97%) were diagnosed with BTT, while 103 patients (66.03%) were diagnosed with IDA. Among patients with BTT, 29 (54.7%) were male, and 24 (45.3%) were female. In contrast, the IDA group was predominantly female, comprising 74 patients (71.8%), with males accounting for 29 patients (28.2%).

Baseline CBC parameters for both groups are summarized in Table 1. Patients with BTT exhibited higher mean RBC counts and lower RDW values compared with those with IDA. Mean Hb and MCV also differed between the two groups, reflecting characteristic hematological distinctions between BTT and IDA.

**Table 1:** Baseline complete blood count parameters Values are expressed as mean ± standard deviation. BTT: beta-thalassemia trait, IDA: iron deficiency anemia, RBC: red blood cell count, Hb: hemoglobin, MCV: mean corpuscular volume, RDW: red cell distribution width, RDWI: red cell distribution width index.

Parameter	BTT (mean ± SD)	IDA (mean ± SD)
RBC (×10⁶/µL)	5.81 ± 0.21	4.52 ± 0.64
Hb (g/dL)	10.33 ± 0.56	8.92 ± 0.88
MCV (fL)	63.85 ± 2.23	71.92 ± 3.86
RDW (%)	11.92 ± 0.47	17.08 ± 2.34
RDWI	131.62 ± 13.71	283.55 ± 74.87
Patra index	5.36 ± 0.13	4.27 ± 0.47

At the cutoff value of ≥5.3 for the Patra index to diagnose BTT, 44 true-positive and 103 true-negative cases were observed, along with 9 false-negative and no false-positive cases. The sensitivity was 83.02% (95% CI: 70.2%-91.9%), and the specificity was 100.00% (95% CI: 96.5%-100.0%). The PPV was 100.00% (95% CI: 92.0%-100.0%), and the NPV was 91.96% (95% CI: 85.3%-96.3%), with a Youden index of 0.83.

Using a cutoff value of <220 for RDWI to diagnose BTT, 53 true-positive and 84 true-negative cases were observed, along with 19 false-positive and no false-negative cases. The sensitivity was 100.00% (95% CI: 93.3%-100.0%), and the specificity was 81.55% (95% CI: 72.7%-88.5%), with a PPV of 73.61% (95% CI: 62.0%-83.2%), an NPV of 100.00% (95% CI: 95.7%-100.0%), and a Youden index of 0.816.

The overall diagnostic efficiency was 94.23% (95% CI: 89.3%-97.3%) for the Patra index and 87.82% (95% CI: 81.6%-92.5%) for RDWI. These findings are summarized in Table 2 and Table 3.

**Table 2:** Diagnostic performance metrics of the Patra index and red cell distribution width index (RDWI) for differentiating beta-thalassemia trait and iron deficiency anemia

Diagnostic parameter	Patra index (≥5.3)	RDWI (<220)
True positives (TP), n	44	53
True negatives (TN), n	103	84
False positives (FP), n	0	19
False negatives (FN), n	9	0
Sensitivity (%)	83.02%	100.00%
Specificity (%)	100.00%	81.55%
Positive predictive value (PPV) (%)	100.00%	73.61%
Negative predictive value (NPV) (%)	91.96%	100.00%
Youden index	0.83	0.816
Diagnostic efficiency (%)	94.23%	87.82%

**Table 3:** Comparative diagnostic performance of the Patra index and red cell distribution width index in differentiating beta-thalassemia trait from iron deficiency anemia, with 95% CI (via the Clopper-Pearson exact method) PPV: positive predictive value, NPV: negative predictive value, RDWI: red cell distribution width index.

Diagnostic parameter	Patra index (≥5.3)	RDWI (<220)
Sensitivity	83.02% (95% CI: 70.2%-91.9%)	100.00% (95% CI: 93.3%-100.0%)
Specificity	100.00% (95% CI: 96.5%-100.0%)	81.55% (95% CI: 72.7%-88.5%)
PPV	100.00% (95% CI: 92.0%-100.0%)	73.61% (95% CI: 62.0%-83.2%)
NPV	91.96% (95% CI: 85.3%-96.3%)	100.00% (95% CI: 95.7%-100.0%)
Diagnostic efficiency	94.23% (95% CI: 89.3%-97.3%)	87.82% (95% CI: 81.6%-92.5%)

The Patra index exhibited superior specificity, PPV, overall diagnostic efficiency, and Youden index compared to RDWI, whereas RDWI demonstrated higher sensitivity.

**Figure 1:**
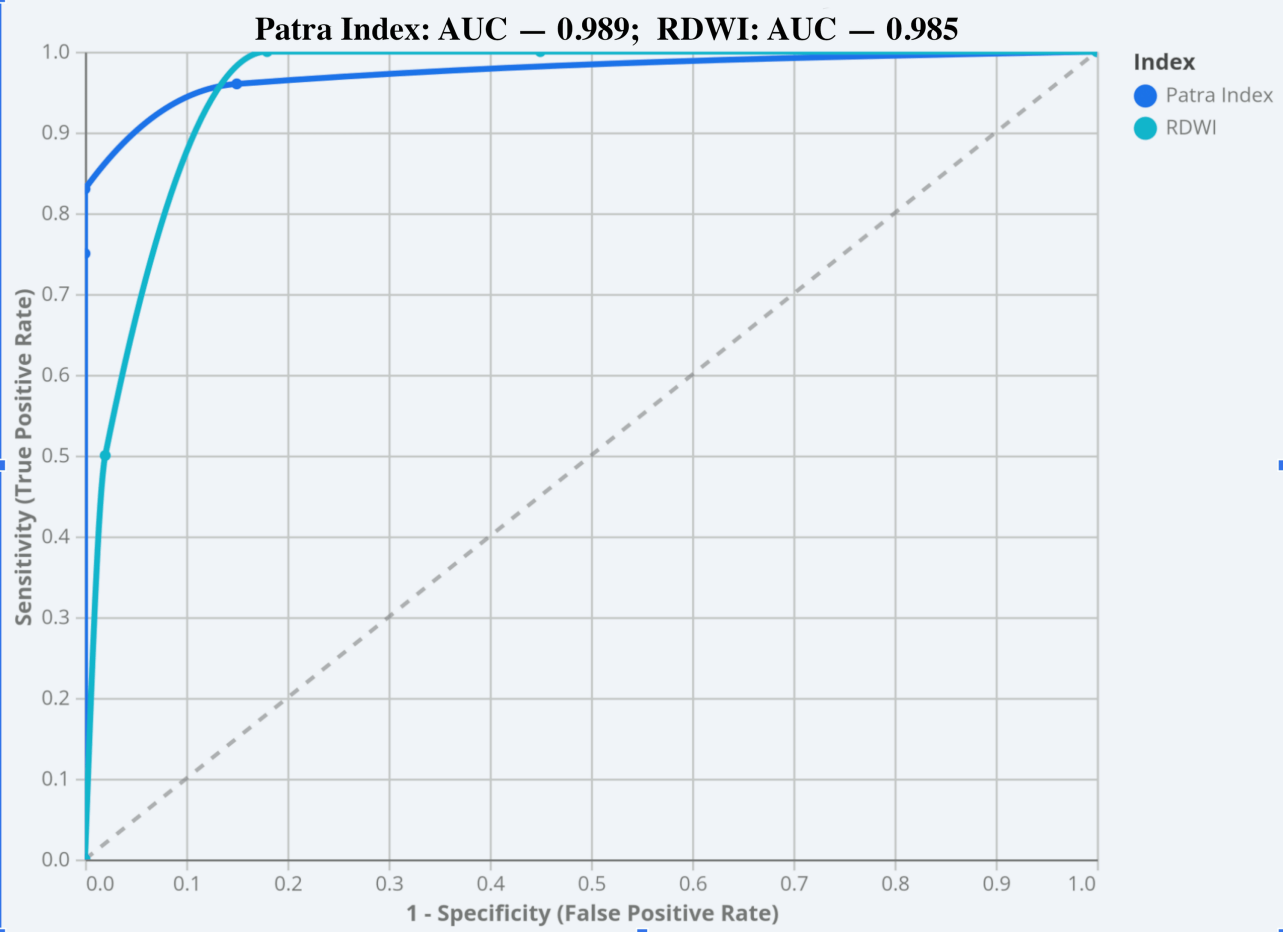
Receiver operating characteristic curve of the Patra index for differentiating beta-thalassemia trait and iron deficiency anemia

ROC curve analysis was performed to evaluate the discriminatory performance of both indices (Figure 1). The Patra index yielded an AUC of 0.989 (95% CI: 0.975-1.000), whereas RDWI showed an AUC of 0.958 (95% CI: 0.924-0.992), both demonstrating high statistical significance (p < 0.0001). The higher AUC and narrower CI for the Patra index indicate superior overall diagnostic performance in distinguishing BTT from IDA.

The cross-classification of index test results against the reference standard is shown in Table 4 and Table 5.

**Table 4:** Cross-classification of the Patra index results against the reference standard diagnosis Values are expressed as number (percentage). Reference standard diagnosis was based on HPLC for BTT and serum ferritin estimation for IDA. A cutoff value of ≥5.3 was used for the Patra index for BTT. BTT: beta-thalassemia trait, IDA: iron deficiency anemia.

Patra index result	BTT (reference +)	IDA (reference −)	Total
Test positive	44 (100.0%)	0 (0.0%)	44 (28.2%)
Test negative	9 (8.0%)	103 (92.0%)	112 (71.8%)
Total	53 (34.0%)	103 (66.0%)	156 (100%)

**Table 5:** Cross-classification of red cell distribution width index results against the reference standard diagnosis Values are expressed as number (percentage). Reference standard diagnosis was based on HPLC for BTT and serum ferritin estimation for IDA. A cutoff value of <220 was used for the RDWI to indicate BTT. BTT: beta-thalassemia trait, IDA: iron deficiency anemia, RDWI: red cell distribution width index.

RDWI result	BTT (reference +)	IDA (reference −)	Total
Test positive	53 (73.6%)	19 (26.4%)	72 (46.2%)
Test negative	0 (0.0%)	84 (100.0%)	84 (53.8%)
Total	53 (34.0%)	103 (66.0%)	156 (100%)

## Discussion

In the present study, the Patra index demonstrated high diagnostic accuracy for differentiating BTT from IDA, with particularly high specificity and overall diagnostic efficiency. The study population predominantly included individuals from a demographic in which both IDA and BTT are highly prevalent [10,11]. In this cohort of biochemically defined BTT and IDA cases, the Patra index performed well; however, cases with overlapping BTT-IDA pathology were not analyzed, and its performance in such mixed cases cannot be inferred. Previous studies have shown that overlapping pathologies may compromise the performance of many traditionally used red cell indices [5,6].

Iron deficiency, the primary cause of IDA in populations with micronutrient deficiencies, leads to increased red cell heterogeneity and elevated RDW, disproportionately affecting indices that rely heavily on RDW or complex combinations of multiple CBC parameters [12,13]. This explains the inconsistent performance of classical discriminant indices, such as the Shine and Lal index, which, despite high sensitivity, often demonstrate low specificity and a high false-positive rate for BTT [5,14,15]. Similarly, indices like Ricerca and Shine and Lal tend to overclassify IDA as BTT due to their sensitivity-weighted design [14-16].

Other indices, including the Srivastava index and England and Fraser index, have shown acceptable specificity but reduced sensitivity in nutritionally challenged populations [15,17,18]. This limitation arises from reliance on Hb and RBC counts, which fluctuate with nutritional status and inflammatory conditions. The Green and King index also demonstrates high specificity but low sensitivity, with inconsistent accuracy across different populations and regions [5,15,19,20]. Likewise, the Kerman index exhibits moderate and variable sensitivity and specificity, limiting its utility as a screening tool [19,21].

More recently proposed indices, such as the CRUISE index, show promising diagnostic accuracy; however, they incorporate multiple parameters with weighted coefficients, which limits ease of use and reproducibility in routine practice, particularly in peripheral healthcare settings [22]. Additionally, the performance of these newer indices has yet to be validated across populations with a high burden of nutritional anemia.

Systematic evaluations, including a meta-analysis by Hoffmann et al., have highlighted that indices such as the microcytic to hypochromic ratio (M/H ratio), Ehsani index, RBC count, and Sirdah index demonstrate high AUC values, whereas commonly used indices like the Mentzer index and RDWI show intermediate performance [19]. Notably, these analyses also emphasize substantial heterogeneity in index performance across age groups and populations, with poorer performance in children and nutritionally challenged cohorts.

Pediatric-specific studies indicate that while the Mentzer index may achieve the highest Youden index, Shine and Lal and Ricerca indices tend to have high sensitivity but low specificity [8,14,16,23]. These findings suggest that indices optimized for sensitivity alone may perform poorly in real-world settings, where a balanced diagnostic accuracy is critical.

In this context, the Patra index appears biologically and methodologically sound. Unlike RDWI, it does not amplify RDW-related variability, and unlike complex indices such as CRUISE, it remains simple and easy to calculate. By retaining sensitivity to microcytosis while attenuating the confounding effects of iron deficiency-induced anisocytosis, the Patra index achieves higher specificity and overall diagnostic efficiency in populations with prevalent iron deficiency. Moreover, it relies on universally reported CBC parameters and does not require analyzer-specific or advanced red cell measurements, making it suitable for implementation in primary health centers and district hospitals with limited laboratory resources.

This study has several limitations. First, the retrospective, single-center design may limit generalizability. Second, detailed baseline clinical variables, such as age stratification, pregnancy status, comorbidities, and recent transfusion history, were inconsistently available and may influence RDW values. Third, while the proposed cutoff of ≥5.3 yielded high specificity, sensitivity remained moderate, which may result in missed BTT cases in high-prevalence regions. Finally, this study utilized a single hematology analyzer, and inter-analyzer variability in RDW and MCV measurements may affect index performance; external validation across diverse laboratory platforms is therefore warranted.

In conclusion, the Patra index may serve as a cost-effective and simple adjunct or triage tool for differentiating BTT from IDA in resource-limited settings. However, it cannot replace confirmatory biochemical testing, including HbA2 estimation. Future prospective studies with well-defined inclusion criteria and broader population representation are necessary to further validate the reliability and diagnostic performance of the Patra index.

## Conclusions

The Patra index is a simple, cost-effective tool that demonstrates high specificity for differentiating biochemically confirmed BTT from IDA in this cohort. However, its moderate sensitivity limits its utility as a standalone screening test, and it cannot replace confirmatory biochemical evaluation. When applied as an adjunct or triage tool, particularly in resource-limited settings, it may help stratify patients and prioritize those requiring further confirmatory testing. Larger, prospective, multicenter studies are needed to validate its generalizability across diverse populations and laboratory platforms.
